# Traumatic fracture of Kager’s fat pad: a case report

**DOI:** 10.1186/s12891-025-09336-x

**Published:** 2025-11-22

**Authors:** Victoria Di Castro Horn, Margaret Hurley, Emily Andrews, Alberto Giardini

**Affiliations:** 1 Philadelphia College of Osteopathic Medicine, 4170 City Line Ave, Philadelphia, PA 19131 USA; 2 Department of Osteopathic Manipulative Medicine, Philadelphia College of Osteopathic Medicine, Philadelphia, PA USA

**Keywords:** Kager's fat pad, Achilles tendon, Pre-calcaneal fat pad, Traumatic injury, Sports medicine

## Abstract

**Background:**

Kager’s fat pad is a metabolically active adipose structure within Kager’s triangle anterior to the Achilles tendon, involved in shock absorption, vascular support, and tendon lubrication. Although most research has focused on its role in Achilles tendinopathy and inflammatory conditions, isolated traumatic disruption without concurrent tendon damage has been rarely documented in the literature, to our knowledge. We report a case of an isolated traumatic fracture of the Kager’s fat pad in the absence of concomitant Achilles tendon injury.

**Case presentation:**

An otherwise healthy 31 year-old female presented to the outpatient musculoskeletal office with reported pain at the distal attachment of the Achilles tendon and the lateral ankle after traumatic injury. The patient was doing a clean and jerk, misplaced the barbell which landed on the patients’ foot, forcing the foot into abrupt supination and dorsiflexion. Musculoskeletal ultrasound (MSK US) revealed a fracture of the Kager’s fat pad without injury to the Achilles tendon.

**Conclusions:**

Our case details the importance of considering pathology of the Kager’s fat pad when investigating posterior ankle pain. Further, it shows the utility of MSK US for the identification of this type of injury, especially in the setting of monetary constraints of patients.

## Background

Kager’s fat pad, also described as the pre-calcaneal or pre-Achilles fat pad, is an adipose structure located superior to the calcaneus in Kager’s triangle. The borders of the triangle include the flexor hallucis longus muscle and tendon anteriorly, the superior cortex of the calcaneus inferiorly, and the Achilles tendon posteriorly [1]. The bursal segment of the pad stretches from the portion between the Achilles tendon and flexor hallucis longus tendon (deep segment of the pad) to the superior tuberosity of the calcaneus, where it forms the anterosuperior border of the retrocalcaneal bursa. Lastly, the posterior segment is adjacent to and anchored by the Achilles tendon [2]. Proposed functions of the Kager fat pad include assisting movement of the bursal wedge during plantarflexion, providing sensory function and protection of the vascular and neural supply to the Achilles tendon, and blunting pressure changes in the retrocalcaneal bursa [3]. Pathology of the Kager fat pad is generally closely associated with Achilles tendinopathy or other pathological processes in adjacent structures (i.e.: calcaneus) [4]. Insertional Achilles tendinopathy occurs at the tendon’s attachment to the calcaneus and often presents with bursal thickening or calcific changes at the insertion, while mid-substance tendinopathy affects the tendon 2–6 cm above the insertion and shows hypoechoic thickening within the tendon body. In this case, the absence of calcaneal tenderness or cortical irregularity, along with an intact tendon on ultrasound, makes either form of tendinopathy unlikely [5]. 

## Case presentation

**Fig. 1:**
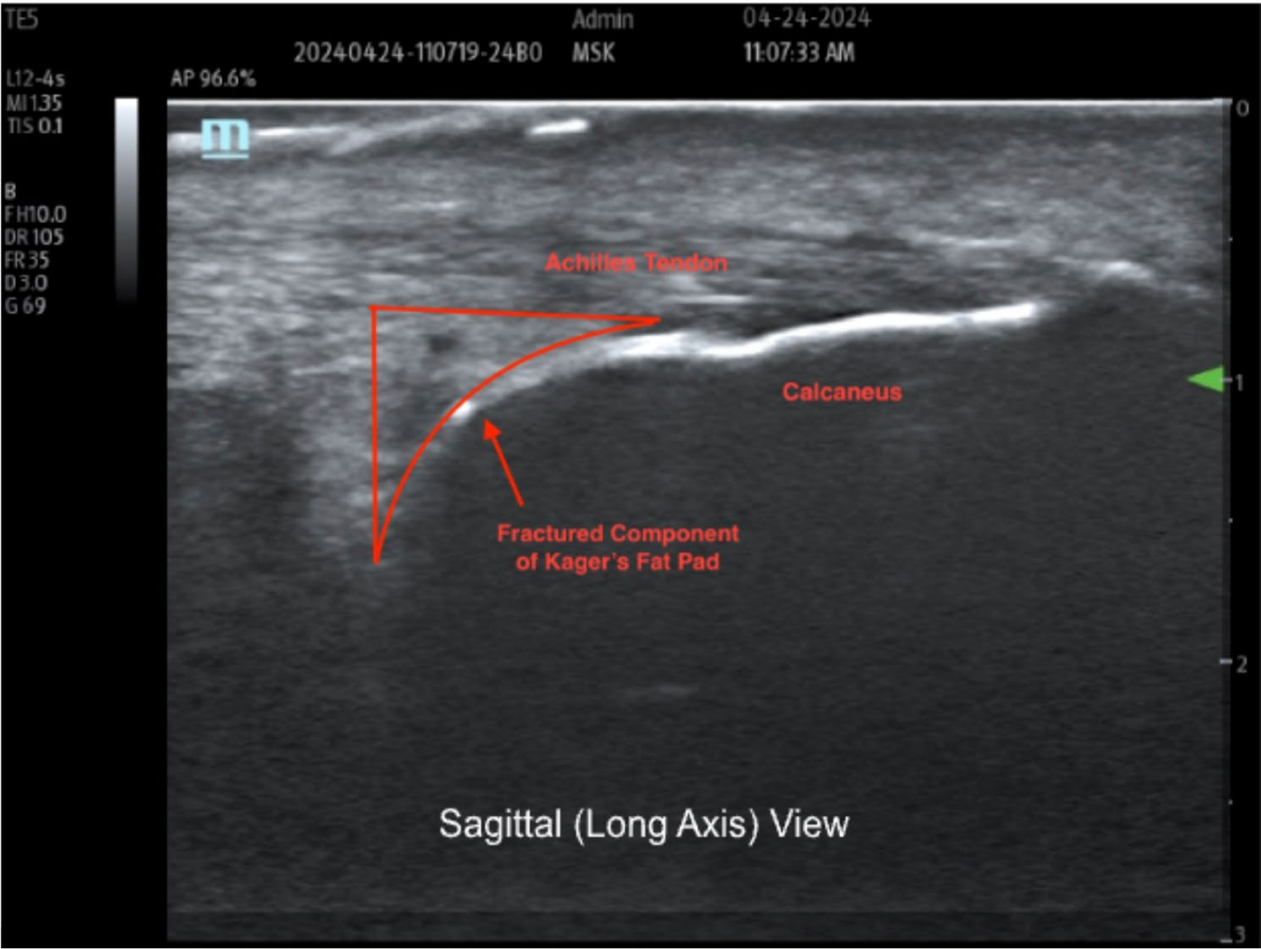
Sagittal plane, neutral (static) Legend: Red outline with arrow showing fractured component of Kager’s fat pad

**Fig. 2:**
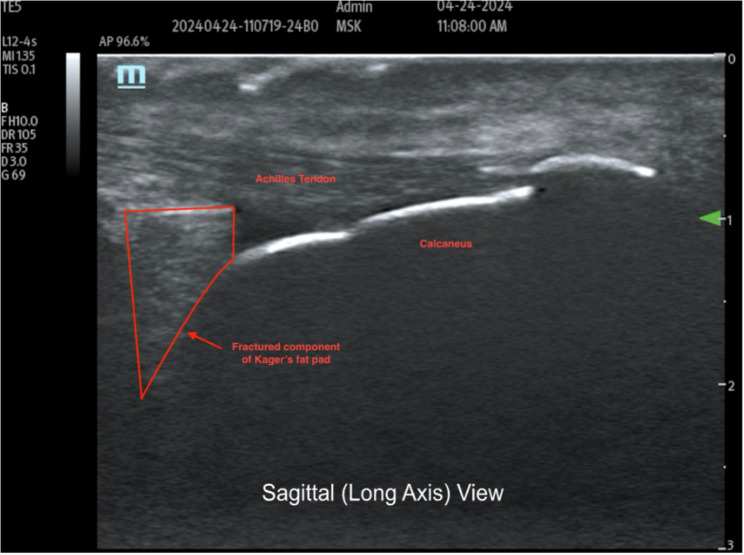
Sagittal plane, dorsiflexion (dynamic) Legend: Fracture and displacement of Kager’s fat pad to the left

**Fig. 3:**
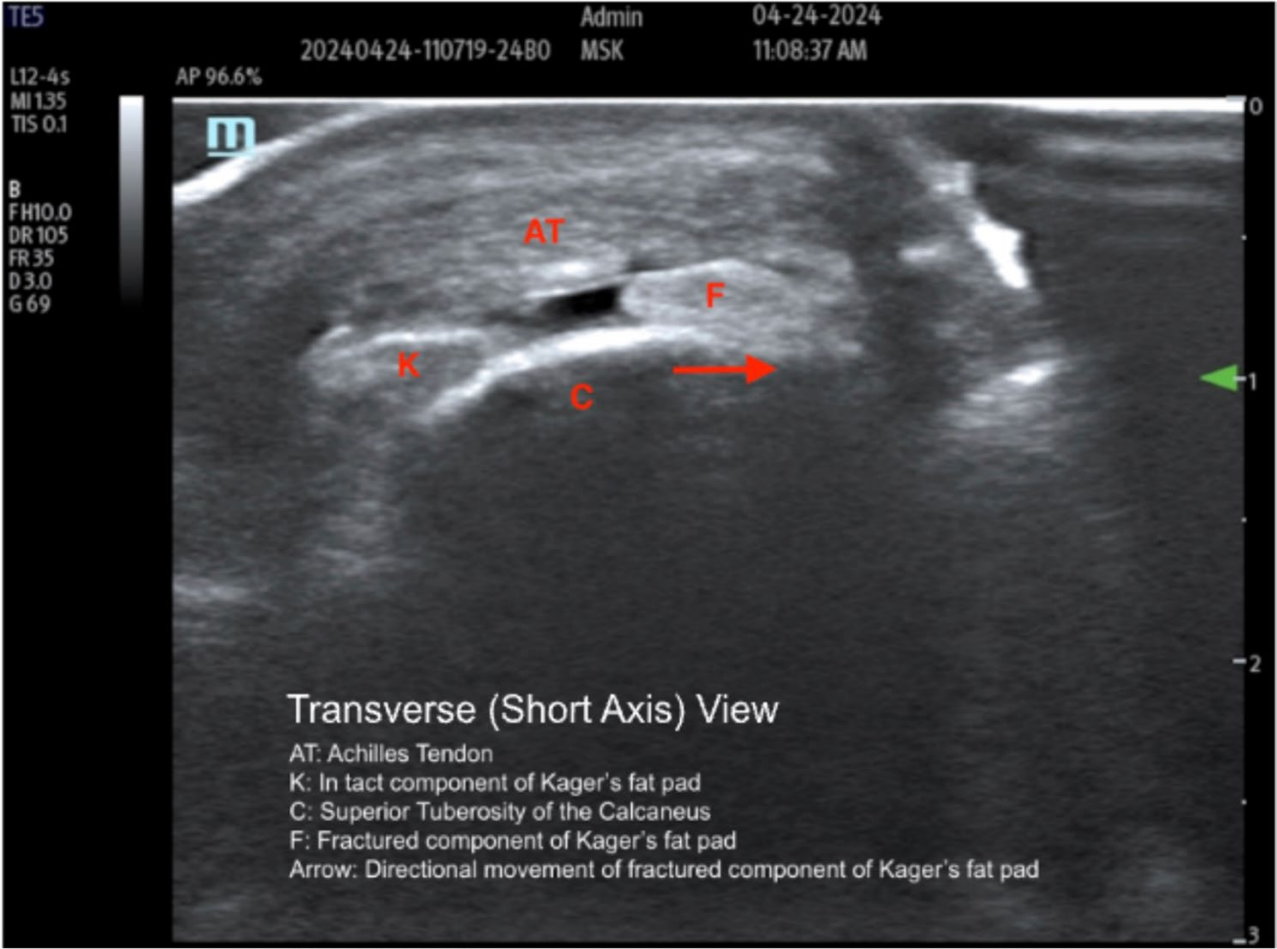
Transverse plane Legend: Demonstrates fracture with hypoechoic space between separated Kager fat pad segments

 A 31-year-old female with no significant past medical history presented to the clinic for evaluation of a 2-week history of right ankle pain and suspected Achilles tendon and anterior talo-fibular ligament (ATFL) injuries. The pain began after an injury sustained while training for Olympic weightlifting. The patient was performing clean and jerks, and while in the overhead split jerk position (right leg posterior with right foot dorsiflexed), failed to stabilize the barbell overhead. The barbell and weight fell onto the right Achilles tendon, causing abrupt and forceful ankle supination followed by dorsiflexion of the foot. The pain was classified as sharp, localized to the distal attachment of the Achilles tendon and the lateral surface of the ankle, and made worse by dorsiflexion of the foot and supination of the ankle. On examination, there was slight edema present around the distal attachment of the Achilles tendon as well as over the ATFL. Tenderness to palpation was present over the Achilles tendon without fusiform thickening. There was no bony tenderness over the calcaneus or other landmarks, no obvious deformity, crepitus, instability suggestive of fracture, or plantar ecchymosis, and the patient did not have weight-bearing issues, making calcaneal fracture unlikely; therefore, radiographs were not performed. Sonographic imaging (Fig. 1) showed a fracture of Kager’s fat pad with an intact Achilles tendon. The patient had no previous history of posterior ankle pain and no other previous traumatic event causing similar pain. The ultrasound was performed using ultrasound in sagittal and axial planes with neutral (static) and active dorsiflexion of the patient’s ankle (Figs. 1, 2 and 3). Magnetic resonance imaging (MRI) was deferred due to financial constraints. Conservative therapy was initiated for our patient, which included physical therapy to improve range of motion and to strengthen surrounding structures. The patient improved enough after 4 weeks of physical therapy to return to activities of daily living, while returning to a full training regimen took several months of conservative therapy.

## Discussion and conclusions

 Pathological injury to Kager’s fat pad has been associated with rheumatoid arthritis, hindfoot abnormalities, calcaneal neoplasms, and Achilles tendinopathy [6]. Achilles tendinopathy (AT) has a prevalence of 2.35 per 1,000 in the general population associated with sporting activities, with elite athletes experiencing this pathology at a lifetime incidence of 24%.[7]. The mechanism of injury experienced by our patient (traumatic fracture of Kager’s fat pad) appears to be uncommon.

Ultrasound imaging is a noninvasive and dynamic imaging modality used to diagnose fractures of Kager’s fat pad [7, 8]. Plain radiographs may only show inflammation or associated calcification and may not show the full breadth of injury. MRI is useful in determining edema within Kager’s fat pad but lacks the dynamic movement which can be visualized using ultrasound [7, 8]. Furthermore, patients are not exposed to radiation as they would be with plain X-ray films, and those with metal pacemakers or other metal prostheses can use ultrasound with no risk [7, 8]. Although MRI is the gold standard for soft tissue imaging, it was not available in this case due to insurance and cost limitations. Nonetheless, dynamic musculoskeletal ultrasound (MSK US) provided high-resolution motion-based visualization that, when interpreted in context, served as a clinically meaningful and cost-effective diagnostic alternative. As supported by recent literature, MSK US can provide rapid correlation with patient symptoms, which allows for immediate dynamic assessment in response to pain or mechanical stress [8]. In cases such as this, where dynamic disruption and mechanical displacement are core components of the working diagnosis, ultrasound may offer comparable diagnostic utility to MRI [1, 7, 8, 11], particularly when performed by a skilled operator, such as in our case, where the diagnostician has five years of experience in MSK US as well as over 200 clinical hours of instruction.

Notably, in Fig. 2, the hyperechoic surface of the calcaneus provides a clear anatomic reference, while the anechoic cleft between the intact and fractured segments of Kager’s fat pad reflects focal separation during dorsiflexion. This space supports a dynamic structural disruption rather than simple post-inflammatory heterogeneity. Further, in the transverse view (Fig. 3), the hyperechoic cortical margin of the calcaneus can be seen, and the central anechoic space seen within Kager’s fat pad reflects a focal cleft created by dynamic separation of the fractured segment. This finding supports true structural disruption rather than diffuse post-inflammatory change.

Due to the unique mechanism of injury reported by the patient and the lack of established protocols for treatment of Kager fat pad injuries, a conservative treatment approach analogous to care for Achilles tendinopathy was chosen. Prevention and care for Achilles tendinopathy includes lifestyle modifications and limitation of activity, physiotherapy, corrective footwear, eccentric loading, and more aggressive management, including platelet-rich plasma and other biological methods [9]. 

Recent evidence underscores the therapeutic significance of Kager’s fat pad in managing mid-portion Achilles tendinopathy. The attachment of the fat pad, being a peritendinous region, signifies it undergoes inflammatory and metabolic changes in the setting of chronic Achilles tendon pathology, which makes this zone a viable target for treatment via injections and other biologics. High-volume injections have proved to be superior for short-term pain relief when compared to platelet-rich plasma or saline alone [10]. Notably, corticosteroid exposure in this zone has been associated with atrophy and fibrosis of Kager’s fat pad [11]. While our case report focuses on the positive outcome of conservative management with rehabilitation, we recognize the utility of injectables in the proper clinical management of similar cases. Future treatment protocols may incorporate image-guided injections, but clinicians should remain alert to iatrogenic changes induced by these therapeutic interventions. Although isolated structural injury to Kager’s fat pad is suspected in this case, such presentations are rare and should be evaluated with caution. When available, dynamic or static MRI may provide additional confirmation and help exclude subtle coexisting pathology. In settings where advanced imaging is not accessible, dynamic MSK ultrasound remains a cost-effective and diagnostically valuable tool, particularly in the hands of experienced clinicians.

This case highlights the diagnostic value of dynamic musculoskeletal ultrasound in detecting rare soft tissue injuries like isolated Kager’s fat pad fracture and emphasizes the importance of considering such pathology in posterior ankle trauma, even in the absence of Achilles tendon involvement.

## Data Availability

No datasets were generated or analysed during the current study.
